# Hierarchical Nanotube-Constructed Porous TiO_2_-B Spheres for High Performance Lithium Ion Batteries

**DOI:** 10.1038/srep11557

**Published:** 2015-07-14

**Authors:** Yi Cai, Hong-En Wang, Shao -Zhuan Huang, Jun Jin, Chao Wang, Yong Yu, Yu Li, Bao-Lian Su

**Affiliations:** 1 State Key Laboratory of Advanced Technology for Materials Synthesis and Processing, Wuhan University of Technology, 122 Luoshi Road, 430070, Wuhan, Hubei, China; 2 Laboratory of Inorganic Materials Chemistry (CMI), University of Namur, 61 rue de Bruxelles, B-5000 Namur, Belgium

## Abstract

Hierarchically structured porous TiO_2_-B spheres have been synthesized via a hydrothermal process using amorphous titania/oleylamine composites as a self-sacrificing template. The TiO_2_-B spheres are constructed by interconnected nanotubes and possess a high specific surface area of 295 m^2^ g^-1^. When evaluated as an anode material in lithium-half cells, the as-obtained TiO_2_-B material exhibits high and reversible lithium storage capacity of 270 mA h g^-1^ at 1 C (340 mA g^-1^), excellent rate capability of 221 mA h g^-1^ at 10 C, and long cycle life with over 70% capacity retention after 1000 cycles at 10 C. The superior electrochemical performance of TiO_2_-B material strongly correlates to the synergetic superiorities with a combination of TiO_2_-B polymorph, hierarchically porous structure, interconnected nanotubes and spherical morphology. Post-mortem structural analyses reveal some discrete cubic LiTiO_2_ nanodots formed on the outer surfaces of TiO_2_-B nanotubes, which might account for the slight capacity loss upon prolonged electrochemical cycling.

Nowadays, a major challenge for rechargeable lithium ion batteries (LIBs) is to develop new materials with high energy density, long cycle life, and excellent rate capability for practical applications in high-power electric vehicles and portable electronic devices^1^. Recently, Titanium-based materials (including TiO_2_, Li_4_Ti_5_O_12_ etc.) have received increasing attention as promising LIBs anode materials due to their low cost, excellent recharge ability, and improved safety over conventional graphite^2^,^3^. Among various polymorphs of TiO_2_ (anatase^4^, rutile^5^, and TiO_2_-B (bronze)^6^), TiO_2_-B shows a favorable channel structure for lithium mobility, resulting in fast charge-discharge capability of LIBs. It has been identified that the lithium intercalation in TiO_2_-B features a pseudocapacitive process, rather than the solid-state diffusion process observed for anatase and rutile^7^. Theoretical studies have revealed that this pseudocapacitive behavior originates from the unique sites and energetics of lithium absorption and diffusion in TiO_2_-B structure^8^,^9^.

Low-dimensional TiO_2_-B nanostructures, such as nanoparticles^10^,^11^, nanowires^12^, nanoribbons^13^,^14^ and nanosheets^15^ have been intensively studied as anode materials in LIBs. In particular, TiO_2_-B nanotubes^16^,^17^, with hollow structure and high surface area, are particularly favorable for electrochemical lithium storage under high rates. However, low-dimensional nanostructures also have some deficiencies, such as easy aggregation, low packing density and poor electrical conduction. In comparison, micro-sized porous secondary particles (e.g., LiFePO_4_^18^, LiMn_2_O_4_^19^, anatase^20^,^21^,^22^,^23^ and rutile^24^,^25^) can overcome these shortcomings yet still maintain the advantages of primary nanomaterials. On the one hand, the stable porous structure can improve electrolyte permeation and decrease polarization resistance. On the other hand, micro-sized secondary particles have better interparticle connection and higher tap density, preventing electrode disintegration during cycling. For instance, Liu *et al.* recently reported the fabrication of nanocrystal-assembled porous TiO_2_-B using titania-silica composite as precursor by a wet-chemical route^26^. For spherical morphology with porous structure, it exhibits superior rate performance.

Therefore, it is highly anticipated that porous TiO_2_-B spheres constructed by interlinked nanotubes can effectively integrate the advantages of TiO_2_–B polymorph, porous structure, 1D nanotubes building blocks and 3D continuous framework. In this case, the interfacial kinetics and intercalation properties of lithium ions could be significantly improved with a combination of pseudocapacitive storage mechanism, adequate electrode-electrolyte contact, and hierarchical robust configuration.

Herein, we report the design and synthesis of hierarchical nanotube-constructed porous TiO_2_-B spheres with high surface area of 295 m^2^ g^-1^, large pore volume of 0.8 cm^3^ g^-1^ and pore size distribution centering around 13 nm. When evaluated as anode materials, the resultant material enables rapid ion and electron transport, resulting in high lithium storage capacity and superior rate capability with 226 mA h g^-1^ at 10 C (1 C equals to 340 mA g^-1^).

## Results

**Figure 1:**
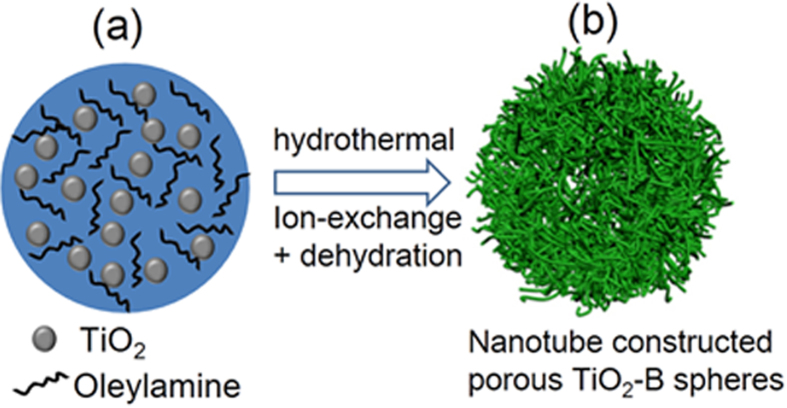
Schematic illustration of the synthetic process of the TiO_2_-B material. (**a**) TiO_2_/oleylamine composite spheres, (**b**) nanotube-constructed porous TiO_2_-B spheres.

**Figure 2:**
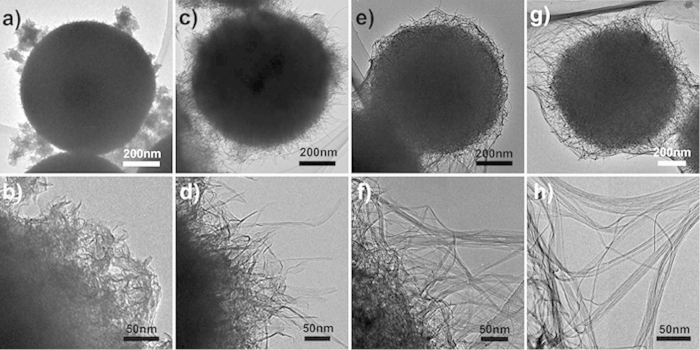
TEM images of sodium titanates samples collected after hydrothermal reactions for different durations. (**a, b**) 15 min, (**c, d**) 1 h, (**e, f**) 12 h, and (**g, h**) 48 h.

Our synthetic strategy is based on an *in-situ* self-templating approach by alkaline hydrothermal transformation of TiO_2_/oleylamine (TO-OA) composite spheres to sodium titanates spheres paired with post-treatments (ion-exchange and dehydration), as schematically illustrated in Fig. 1. In the first step, amorphous TO-OA composite spherical particles were synthesized by a modified sol-gel route^27^ and used as a self-sacrificing template (Fig. 1a and Supplementary Fig. S1a). Next, hierarchical sodium titanates (Na-titanates) spheres were obtained by hydrothermal treatment of the TO-OA precursors in an alkaline solution. During this process, the OA molecules contained in the composite spheres were dissolved out in ethanol and sodium ions (Na^+^) were inserted into the amorphous TiO_2_ along with breakage of some Ti-O bonds and formation of Na-titanates^28^. The morphology and structure evolution of the Na-titanates have been studied by analyzing a series of intermediate products harvested after different durations. Transmission electron microscopy (TEM) image indicates that, after hydrothermal reaction for 15 min, the surface of the spheres becomes rough and some lamellar fragments are formed (Fig. 2a,b). When prolonging reaction time to 1 h, the lamellar fragments around the spheres gradually evolve into nanosheets (Fig. 2c,d). After 12 h of reaction, some nanotubes are formed, as shown in Fig. 2e,f. The formation of nanotubes can be ascribed to the scrolling of lamellar nanosheets to reduce total surface energy of reaction system^29^,^30^. Further extending hydrothermal time to 48 h, porous Na-titanates spheres comprising connected nanotubes are obtained (Fig. 2g,h). X-ray diffraction (XRD) pattern (Supplementary Fig. S2) indicates that the Na-titanates sample obtained after reaction for 48 h has an orthorhombic Na_2_Ti_2_O_5_·H_2_O phase. After ion-exchange, the resultant protonated titanate (H_2_Ti_2_O_5_·H_2_O) sample retains orthorhombic structure (JCPDS Card No. 47-0124)^28^.

**Figure 3:**
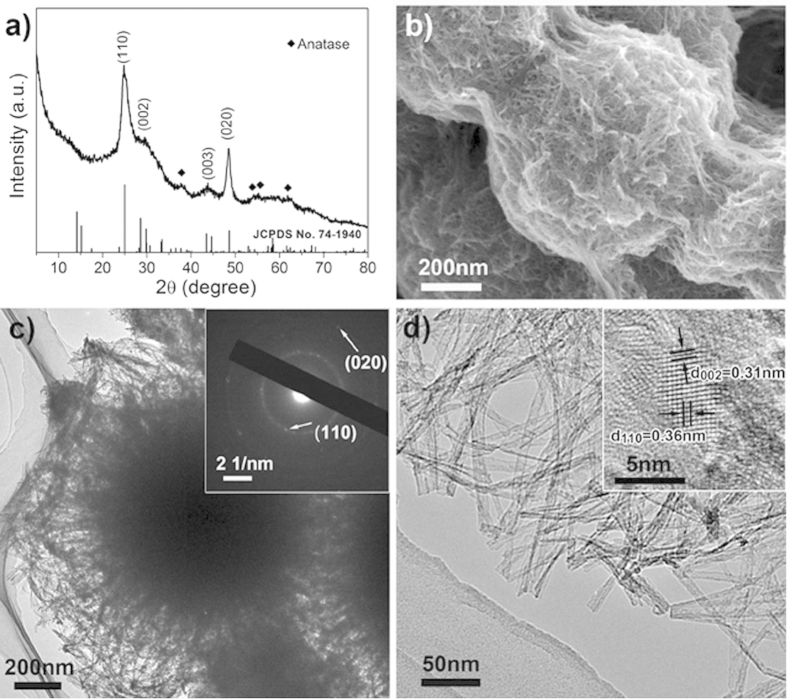
(**a**) XRD pattern, (**b**) SEM image, (**c**) TEM micrograph, SAED pattern (inset), (**d**) high-magnification TEM and HRTEM (inset) of the as-prepared porous TiO_2_-B spheres constructed by nanotubes.

The protonated titanates were transformed to hierarchical nanotube-constructed porous TiO_2_-B spheres via dehydration at 300 ^o^C for 2 h in air (Fig. 1b). Figure 3a shows the XRD pattern of the prepared TiO_2_ material, in which the majority of intense diffraction peaks can be indexed to monoclinic TiO_2_-B phase (JCPDS Card No. 74-1940)^26^. The large half-peak width indicates the low crystallinity of the as-obtained TiO_2_-B material. In addition, some minor peaks can be identified as tetragonal anatase phase (JCPDS Card No. 21-1272)^31^, suggesting partial transformation of TiO_2_-B to anatase during heat treatment, which coincides with some previous reports^26^,^32^.

The morphology and microstructure of the as-prepared materials were analyzed by electron microscopy characterization methods. Scanning electron microscopy (SEM) image (Supplementary Fig. S1b) shows the TiO_2_-B sample retains its overall spherical morphology when compared to the precursor TO-OA material; however, the structure has become highly porous. A careful inspection shown in Fig. 3b reveals that each porous sphere has a particle size of ~500 nm and consists of a large number of TiO_2_-B nanotubes. These nanotubes are highly interconnected and form a 3D porous structure, which might possess enhanced electronic conduction due to the accumulation of electrons at the sintered grain-grain interfaces. Energy-dispersive X-ray (EDX) spectrum (Supplementary Fig. S3) indicates the presence of titanium, oxygen, and carbon elements in the as-obtained material. The existence of carbon may be attributed to the carbonization (incomplete combustion) of residual OA molecules adsorbed on the particle surface. Thermogravimetric analysis (TGA) and differential scanning calorimetry (DSC) were further performed in air to determine the carbonaceous content of the as-obtained TiO_2_-B material. As shown in Supplementary Fig. S4, two distinct regions of weight loss can be observed. The initial weight loss around 3% before 200 °C corresponds to the physically adsorbed water in the sample. The second weight loss about 7.1% between 200 and 400 °C, with a big exothermic peak, denotes the decomposition of carbonaceous substances. The presence of carbon in the as-obtained TiO_2_-B material might also contribute to the improved lithium storage capability due to the possibly increased electrical conduction and structural stability as will be discussed in the following.

Transmission electron microscopy (TEM) micrograph and selected-area electron diffraction (SAED) pattern (Fig. 3c and its inset) further confirm the interlinked tubular TiO_2_-B nanostructures. High-magnification TEM micrograph (Fig. 3d) clearly reveals that the TiO_2_-B nanotubes typically have diameter of *ca.* 10 nm and wall thickness of *ca.* 2 nm. The clear lattice fringes with interplanar spacings of 0.36 nm and 0.31 nm shown in high-resolution TEM (HRTEM) micrograph can be well ascribed to the (110) and (002) crystal planes of monoclinic TiO_2_-B, respectively. These comprehensive results clearly validate that the as-prepared material combines the superiorities of TiO_2_-B polymorph, nanotube structure and porous spherical morphology.

The hierarchical nanotubular porous structures may endow the as-synthesized TiO_2_-B material with a high surface area. Nitrogen adsorption/desorption isotherms were measured to determine the specific surface area and porosity of the TiO_2_-B material. As shown in Supplementary Fig. S5, the isotherms curve shows a typical Type IV isotherm representing mesoporous structure with a Brunauer-Emmett-Teller (BET) specific surface area of 295 m^2^ g^-1^ and pore volume of 0.8 cm^3^ g^-1^. The pore size distribution shown in the inset of Supplementary Fig. S5 displays a pore distribution centering around 13 nm.

**Figure 4:**
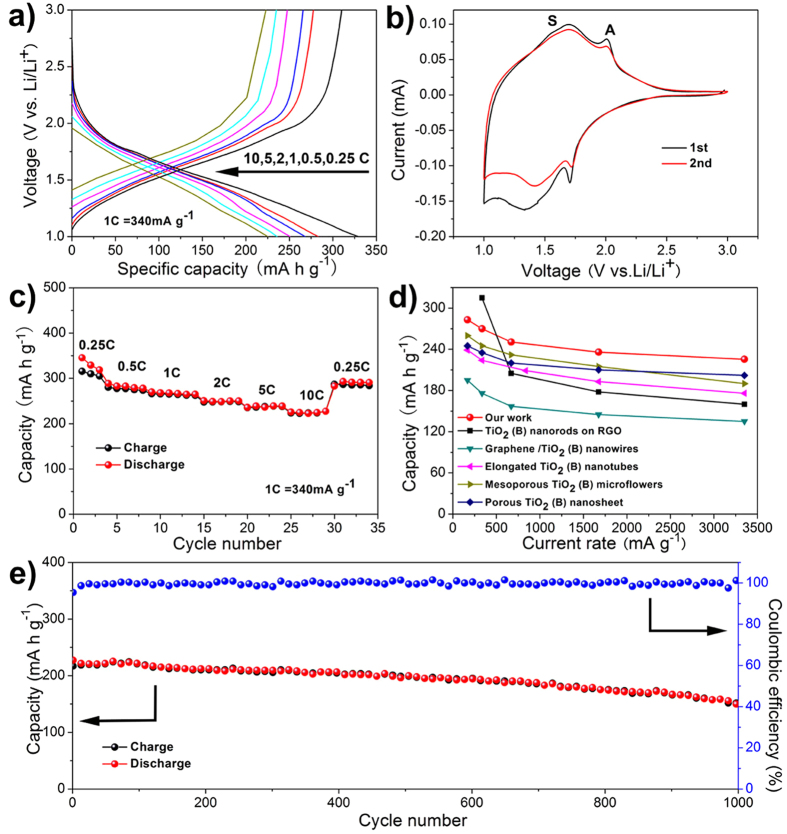
Electrochemical properties of the TiO_2_-B material. (**a**) galvanostatic discharge-charge curves at different current rates; (**b**) the first two CV curves at a sweep rate of 0.2 mV s^-1^; (**c**) rate performance; (**d**) comparison of rate capability between this work and other previously reported works at various current densities; (**e**) cycling performance at high current rate of 10 C over 1000 cycles.

As mentioned above, such unique structure may exhibit superior lithium storage performance. The electrochemical performance of porous TiO_2_-B spheres was evaluated in lithium half-cells. As shown in Fig. 4a, the discharge curve at low rate (0.25 C) shows a plateau at *ca.* 1.7 V and a sloped region of 1.7–1.0 V, corresponding to the lithiation of anatase and pseudocapacitive process of low-crystallized TiO_2_-B phase, respectively. The TiO_2_-B material at 0.25 C delivers a high lithiation capacity of 327 mA h g^-1^, which is near to its theoretical capacity (340 mA h g^-1^) and a de-lithiation capacity of 310 mA h g^-1^. The potential plateau of anatase at 1.7 V gradually diminishes with increasing current rates from 0.5 C to 10 C, indicating that the TiO_2_-B phase contributes major part of the capacity, especially at higher current rates. Figure 4b depicts the first two cyclic voltammetry (CV) curves of the TiO_2_-B electrode at a sweep rate of 0.2 mV s^-1^. The redox peaks located around 1.5 and 1.7 V (denoted as S-peaks) are the characteristic pseudocapacitive behavior of lithium storage in TiO_2_-B. The absence of splitting of S-peaks can be ascribed to the low-crystallinity of the as-obtained TiO_2_-B material. In addition, a pair of peaks located between 1.6 V and 2.2 V (denoted as A-peaks) is the typical behavior expected for solid-state diffusion of lithium intercalation in anatase. Figure 4c shows the charge-discharge capacities of porous TiO_2_-B spheres at different current rates. For comparison, TiO_2_-B nanoribbons with diameters of 100–200 nm and lengths up to 10 μm (Supplementary Fig. S6) and commercial Degussa P25 nanoparticles were also evaluated under similar testing conditions. Clearly, the porous TiO_2_-B spheres deliver higher cycling capacity than the others, especially at higher current rates. It offers a high capacity of 221 mA h g^-1^ even at 10 C, which is much better than those performance of TiO_2_-B nanoribbons (93 mA h g^-1^ at 5 C) and commercial P25 nanopowders (62 mA h g^-1^ at 5 C), respectively (Supplementary Fig. S7). The high-rate performance of TiO_2_-B material would be attributed to its characteristic pseudocapacitive behavior for fast lithium storage. On the contrary, commercial P25 nanopowders show a dramatic capacity drop at high current densities due to the slow lithium diffusion in solid-state phase and the resultant strong polarization as well as self-aggregation during the electrode fabrication and electrochemical cycling process. Compared to TiO_2_-B nanoribbons, the small diameter and tube wall thickness of the nanotubes shorten the diffusion lengths of lithium ions. Moreover, the high specific surface area of the porous material provides large electrode-electrolyte contact area for rapid electrochemical reactions. In addition, the electrochemical performance of our material is superior to those of several TiO_2_-B nanostructure (Fig. 4d), such as TiO_2_-B nanorods on reduced graphene oxide (RGO)^33^, graphene/TiO_2_-B nanowires^34^, elongated TiO_2_-B nanotubes^17^, mesoporous TiO_2_-B microflowers^35^, and porous TiO_2_-B nanosheets^32^.

**Figure 5:**
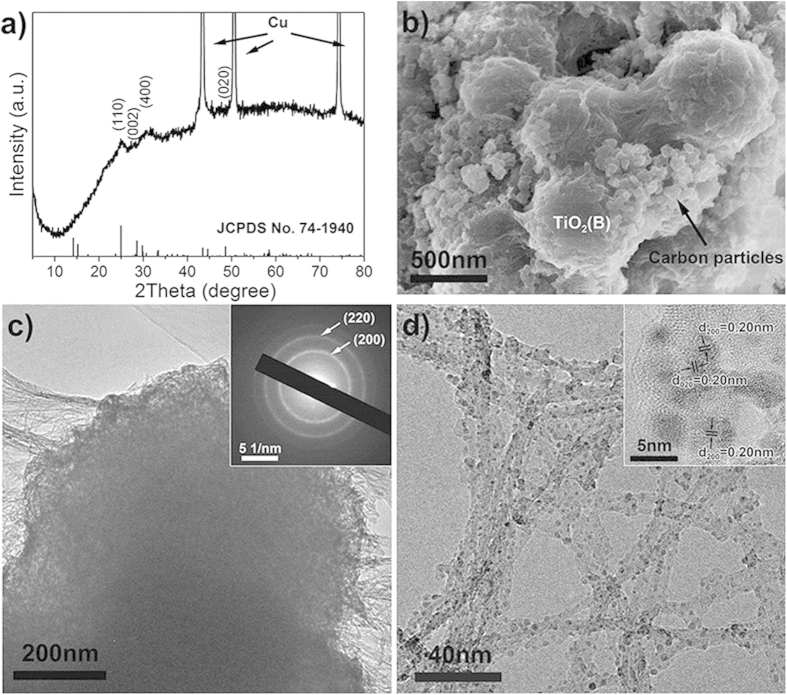
(**a**) XRD pattern, (**b**) SEM image, (**c**) TEM micrograph, SAED pattern (inset), (**d**) high-magnification TEM and HRTEM (inset) of the TiO_2_-B material after discharge-charge measurements at 10 C for 1000 cycles.

In order to test the cyclability at high rates, a lithium cell using porous TiO_2_-B spheres was run at 10 C (3400 mA g^-1^) for 1000 cycles. Figure 4e shows the capacity starts at 221 mA h g^-1^ and maintains at 211 mA h g^-1^ over 200 cycles, with a capacity loss of 4.5%, which is superior to the nanosheet-constructed porous TiO_2_-B spheres (7.4%)^32^. Even after 1000 cycles, the reversible capacity retains 154 mA h g^-1^ with a capacity loss of 30%. The superior cycling performance certainly testifies the high stability of the porous structure and the good accommodation to volume/strain changes during lithium insertion-extraction. The structural details of the TiO_2_-B material after extended cycling at high current rate were further studied by CV measurement and physical characterizations, as shown in Supplementary Fig. S8 and Fig. 5. The CV curves measured at varying sweep rates clearly validate the pseudocapacitive lithium storage of TiO_2_-B as well as the existence of trace anatase phase. From Fig. 5a, the majority of diffraction peaks can be indexed to monoclinic TiO_2_-B phase in addition to three strong peaks arising from the Cu current collector. The SEM image shown in Fig. 5b reveals the spherical shape of the hierarchical nanotube-constructed TiO_2_-B particles has been well preserved, suggesting the high structural stability of the as-prepared TiO_2_-B material. In addition, the conductive carbon particles additives with a size of ~50 nm are found only attaching on the outer surfaces of the TiO_2_-B spheres. This result indirectly proves the hierarchical nanotube-constructed porous TiO_2_-B spheres have an improved electronic conduction capability and reduced polarization. TEM micrographs (Fig. 5c,d) further reveal that the porous spherical structure has been well preserved and the nanotubes remain highly interconnected after electrochemical cycling, suggesting the high structural stability. The 3D well interconnection among the nanotubes as well as the existence of carbon within the hierarchical TiO_2_-B spheres should contribute largely to their improved electronic conduction and structural stability. In addition, it is noteworthy that some nanodots evolved on the surface of nanotubes, which are different from the as-obtained pristine TiO_2_-B material before electrochemical tests. The SAED pattern taken from the TEM micrograph (inset of Fig. 5c) and the lattice fringes in HRTEM micrograph (inset of Fig. 5d) prove that the newly formed nanodots can be indexed to cubic LiTiO_2_ (space group: Fm3m, JCPDS Card No. 74-2257). Recent studies have shown that amorphous TiO_2_ nanotubes prepared by electrochemical anodization can gradually convert into cubic lithium titanate during electrochemical tests^36^. It remains unclear herein whether these LiTiO_2_ nanodots stemmed from the amorphous region of our low-crystallization TiO_2_-B material upon prolonged discharge-charge cycling. However, the observation of these isolated cubic LiTiO_2_ nanodots can explain the full lithiation capability of our TiO_2_-B material and possible reason for capacity fading after long-term electrochemical cycling. Further work is in progress to fully understand the possible formation mechanism of these LiTiO_2_ nanodots and their role in affecting the electrochemical performance of TiO_2_-B.

## Discussion

We designed and synthesized hierarchically structured porous TiO_2_-B spheres constructed by interconnected nanotubes, which combined the advantages of TiO_2_-B polymorph, porous morphology and ultrathin nanotubes. The TiO_2_-B polymorph ensures fast insertion and extraction kinetics of lithium ions due to its favorable open channel structure. The porous structure with interconnected nanotubes effectively results in adequate electrode-electrolyte contact, reduced ionic diffusion path, and facile electronic transport along the 3D network. The integration of these features endows this material superior lithium storage performance, which could meet the needs of next-generation high-power rechargeable batteries.

## Methods

### Synthesis of porous TiO_2_-B

All the reagents were analytical pure grade and used without any further purifications. The synthesis protocol of amorphous TiO_2_/oleylamine (TO-OA) composite spheres was adapted from recent literature^27^ with little modification. In a typical procedure, 4.5 mL tetrabutyl titanate was added into 200 mL ethanol (EtOH) containing 1.8 mL oleylamine (OA) and 0.8 mL H_2_O under vigorous stirring at ambient temperature. The resulting white precipitates were kept static at the room temperature overnight, then collected by centrifugation and washed with EtOH three times and finally dried at 80 °C in air for 12 h.

The sodium titanate spheres were prepared by hydrothermal treatment of 0.2 g of TO-OA precursors in a EtOH/H_2_O mixture (40 and 20 mL respectively) containing 12 g of sodium hydroxide (NaOH) in a 100 mL Teflon-lined autoclave at 150 °C for 48 h. After that, the white precipitates were rinsed with ethanol and distilled water in sequence, and finally dried at 80 °C in air for 12 h.

To prepare hierarchical porous TiO_2_-B spheres, the sodium titanates were transformed into protonated titanates by rinsing in 200 mL of 0.12 M hydrolytic acid (HCl) solution for 2 h with ion-exchange (Na^+^ with H^+^), dried and then dehydration at 300 °C for 2 h in air.

### Materials characterization

Powder X-ray diffraction (XRD) patterns were recorded on a Bruker diffractometer with Cu K*α* radiation (λ = 1.54056 Å) at 40 kV/mA. The morphology and particle size of the samples were observed by field-emission scanning electron microscope (SEM, Hitachi S-4800) at an acceleration voltage of 5 kV. Energy-dispersive X-ray spectroscopy (EDX) was performed using an EDAX Genesis instrument attached to SEM with an acceleration voltage of 30 kV. Transmission electron microscopy (TEM) and high-resolution TEM (HRTEM) were performed on a JEOL JEM-2100F microscope with an acceleration voltage of 200 kV. Nitrogen adsorption/desorption isotherms were acquired using a Micrometrics, TriStar II 3020 system operated at 77 K. Prior to the adsorption experiments, the samples were degassed at 100 °C for 12 h. Thermogravimetric analysis (TGA) and differential scanning calorimetry (DSC) curves of the as-fabricated materials were performed using a Labsys EvoS60/58458 thermal analysis instrument at a temperature ramping rate of 5 °C min^-1^ in air.

### Electrochemical measurements

The working electrodes were fabricated by using active materials (TiO_2_), conductive carbon blacks (Super-P) and polyvinylidene fluoride (PVDF) binder in a weight ratio of 80: 10: 10. The slurry was coated on a copper foil and dried in a vacuum oven at 100 °C for 12 h. Electrochemical measurements were carried out via CR2025 coin type cells using lithium foils as the counter electrode and reference electrode, a 1 M solution of LiPF_6_ dissolved in ethylene carbon (EC)/dimethyl carbonate (DMC) (1:1 w/w) as electrolyte. The lithium cells were assembled in an argon-filled glove-box with both water and oxygen contents below 1 ppm. Cyclic voltammetry (CV) measurements were carried out using a CHI 604e electrochemical workstation at different sweep rates. Galvanostatic charge/discharge cycling experiments were carried out on a multichannel battery testing system (LAND CT2001A, Wuhan, China) in a potential range of 1 – 3 V vs. Li/Li^ + ^. All the electrochemical tests were carried out at room temperature (25 °C).

## Additional Information

**How to cite this article**: Cai, Y. *et al.* Hierarchical Nanotube-Constructed Porous TiO_2_-B Spheres for High Performance Lithium Ion Batteries. *Sci. Rep.* **5**, 11557; doi: 10.1038/srep11557 (2015).

## Supplementary Material

Supplementary Information
