# Supplementary material for: Hierarchical Nanotube-Constructed Porous TiO2-B Spheres for High Performance Lithium Ion Batteries
Source: Sci Rep. 2015 Jul 14;5:11557. doi: 10.1038/srep11557 (PMC4501008; doi:10.1038/srep11557)
Supplement: Supplementary Information [file srep11557-s1.doc]

**Supplementary Information**

**Hierarchical Nanotube-Constructed Porous TiO_2_-B Spheres for High Performance Lithium Ion Batteries**

Yi Cai^1^, Hong-En Wang^1,*^, Shao-Zhuan Huang^1^, Jun Jin^1^, Chao Wang^1^, Yong Yu^1^, Yu Li^1^ & Bao-Lian Su^1,2,*^

^1^State Key Laboratory of Advanced Technology for Materials Synthesis and Processing, Wuhan University of Technology, 122 Luoshi Road, 430070, Wuhan, Hubei, China; Fax: +86 27 87879468; Tel: +86 27 87855322

^2^Laboratory of Inorganic Materials Chemistry (CMI), University of Namur, 61 rue de Bruxelles, B-5000 Namur, Belgium; Fax: +32 81 725414; Tel: +32 81 724531

* [*hongenwang@whut.edu.cn*](mailto:hongenwang@whut.edu.cn) and [*bao-lian.su@unamur.be*](mailto:bao-lian.su@unamur.be)


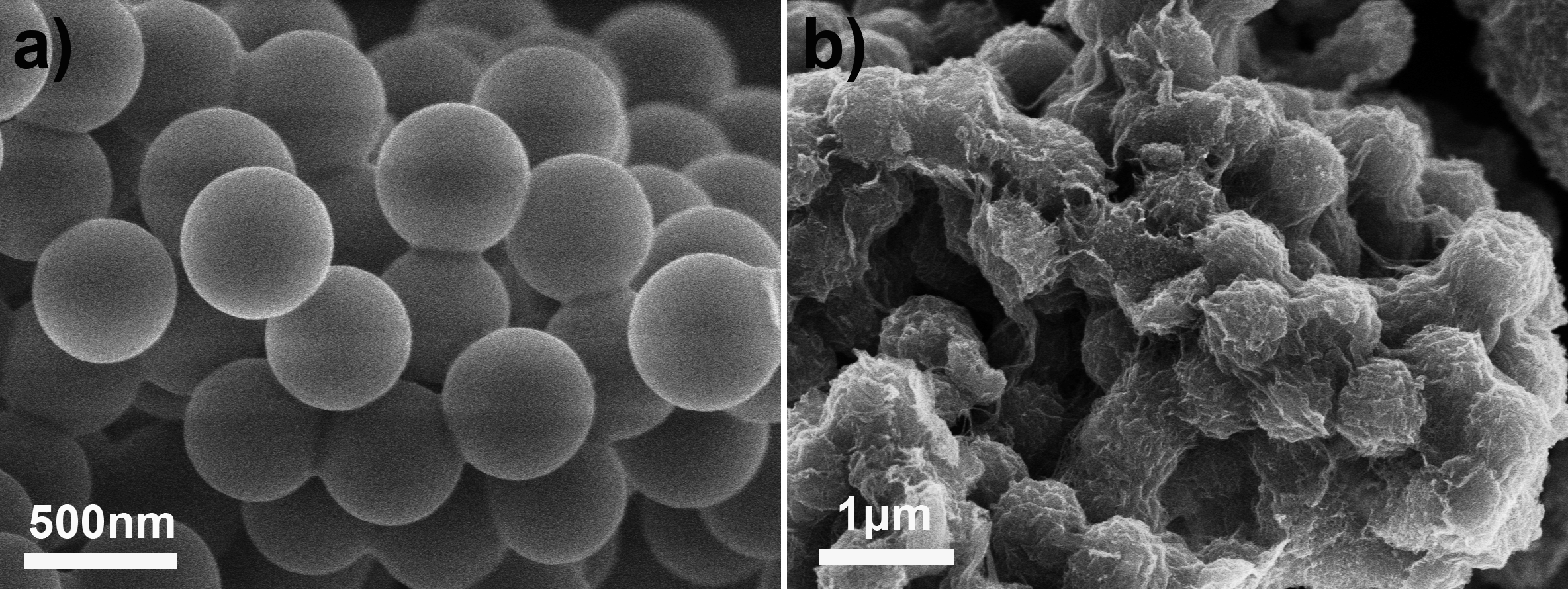


**Figure S1.** SEM images of (a) the TiO_2_/oleylamine composite spheres and (b) low-magnification micrograph of the as-prepared TiO_2_-B material.


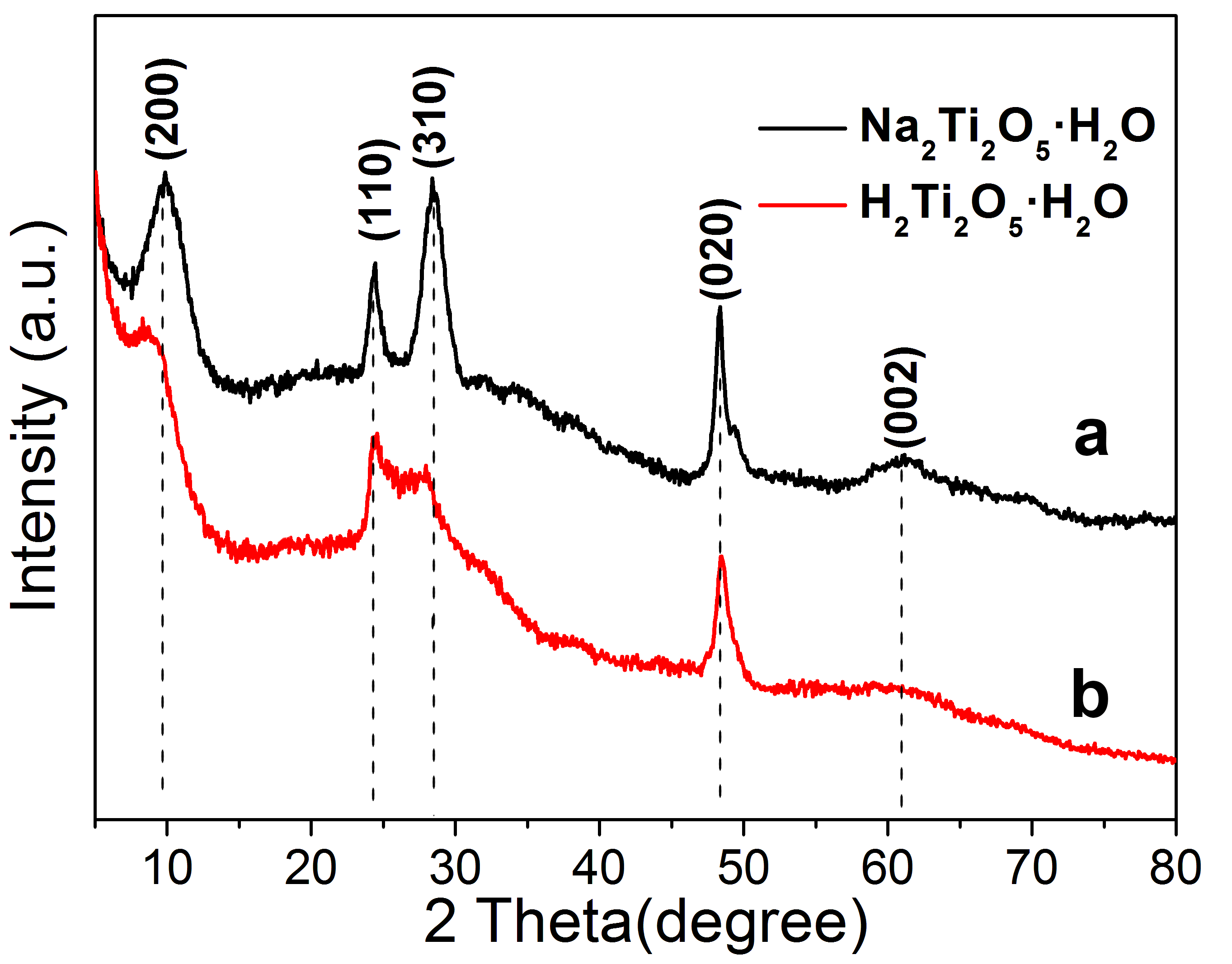


**Figure S2.** XRD patterns of the as-prepared sodium titanates and protonated titanates. The diffraction peaks located at 9.78°, 24.06°, 27.86° and 48.02° correspond to the (200), (110), (310) and (020) planes of H_2_Ti_2_O_5_·H_2_O (JCPDS Card No. 47-0124). The crystal structure of Na_2_Ti_2_O_5_·H_2_O is similar to that of H_2_Ti_2_O_5_·H_2_O.


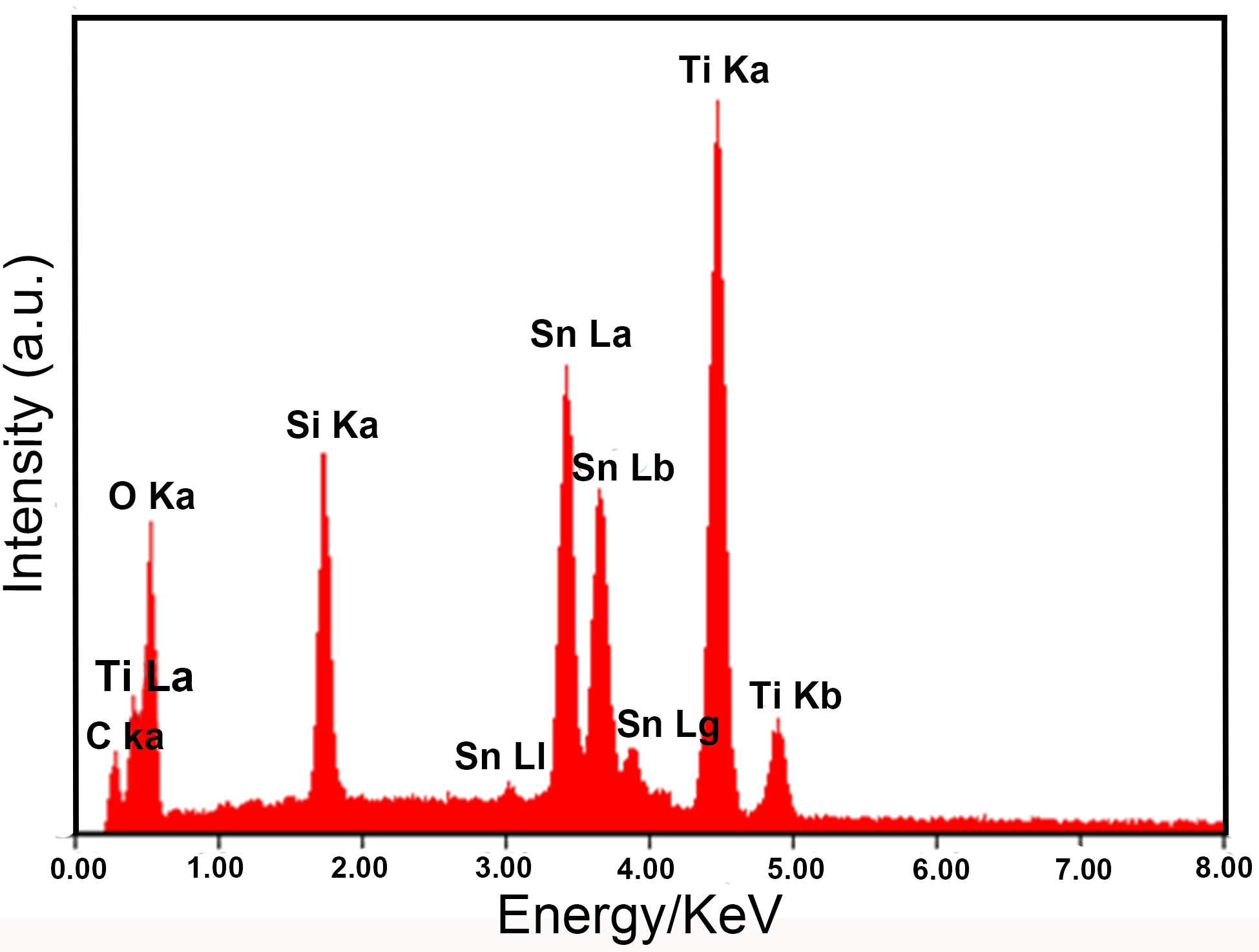


**Figure S3.** EDX pattern of the as-prepared TiO_2_-B material. The tin and silicon elements, and part of oxygen signal originate from the fluorine-doped tin oxide (FTO)-glass substrate.


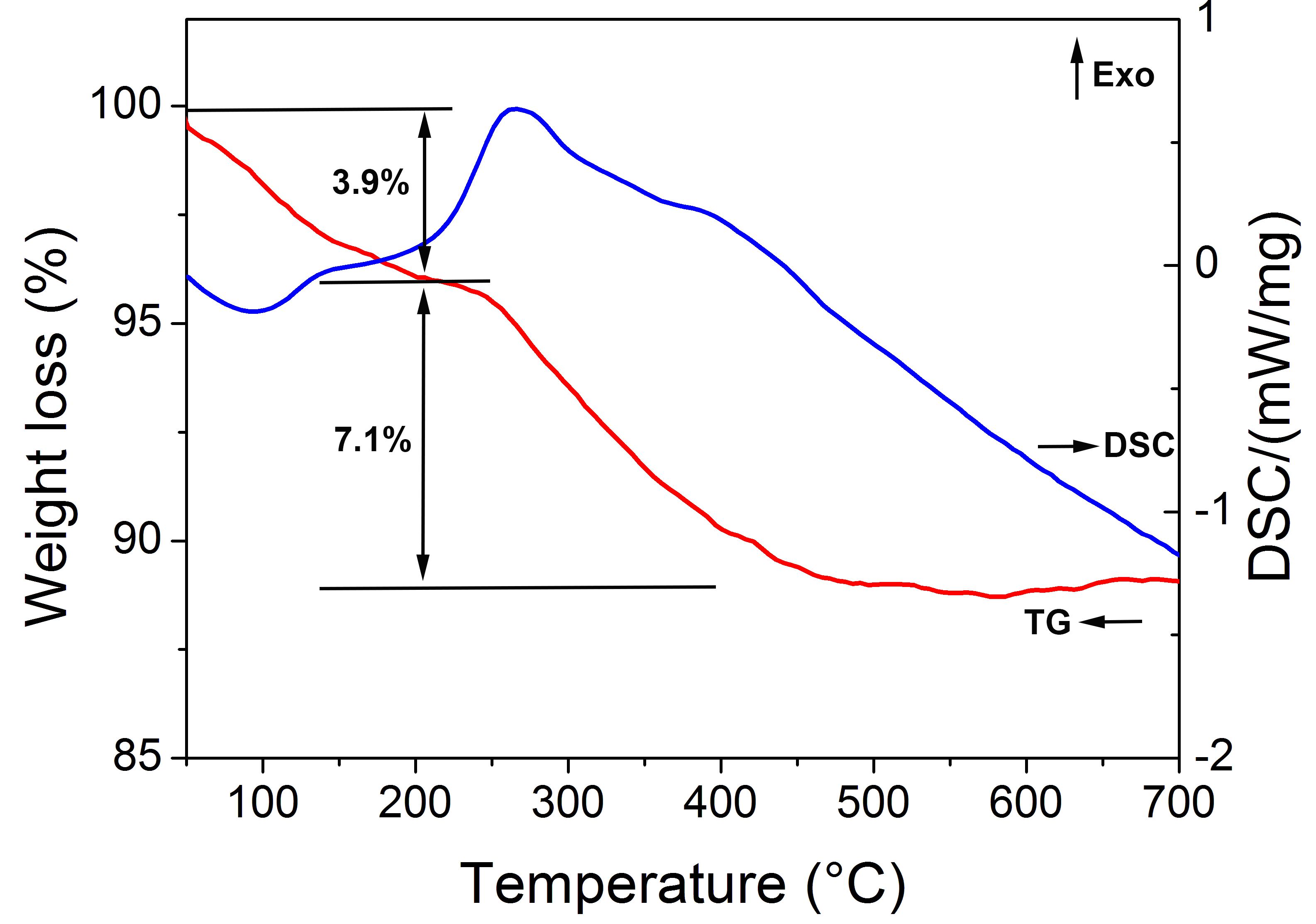


**Figure S4.** TG/DSC plots of the as-prepared TiO_2_-B material in air at a temperature ramping rate of 5 °C min^-1^.


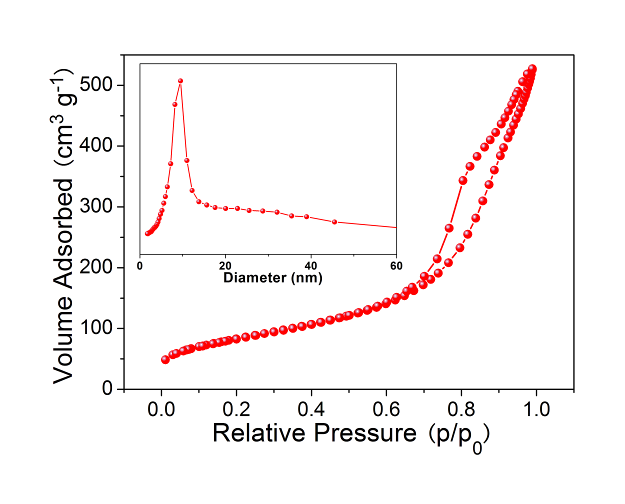


**Figure S5.** Nitrogen adsorption/desorption isotherms and pore size distribution curve (inset) of the hierarchical nanotube-constructed porous TiO_2_-B spheres.


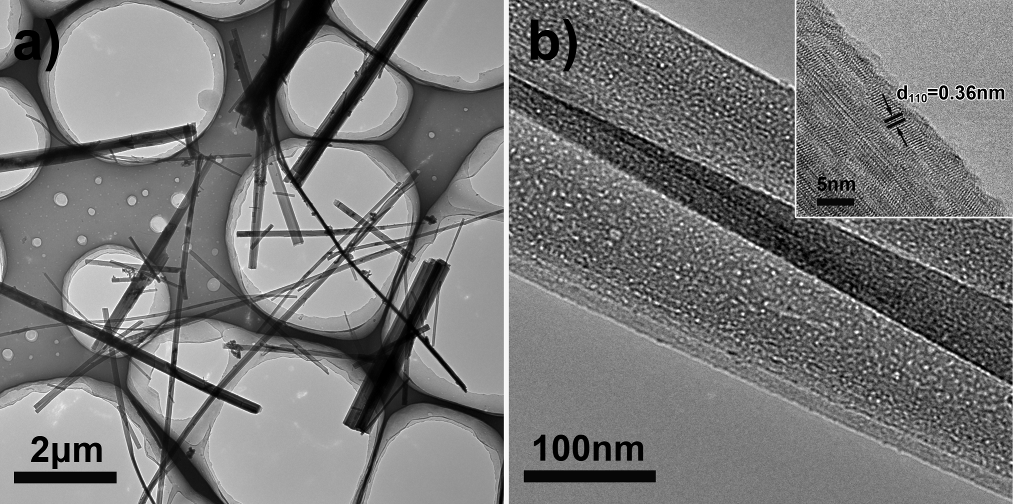


**Figure S6.** (a) TEM micrograph, (b) high-magnification TEM and HRTEM micrographs (inset) of TiO_2_-B nanoribbons.


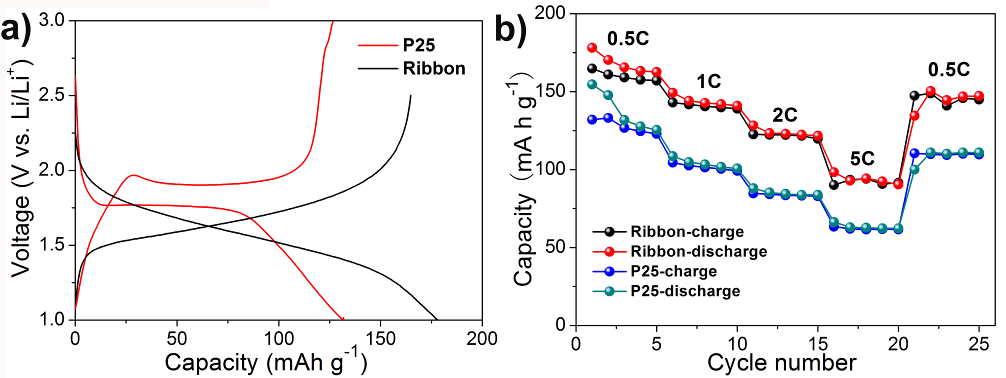


**Figure S7.** Electrochemical properties of TiO_2_-B nanoribbon with disconnected cavities and commercial P25 nanopowders for reference. (a) discharge-charge curves at 0.2 C (68 mA g^-1^) and (b) rate properties.


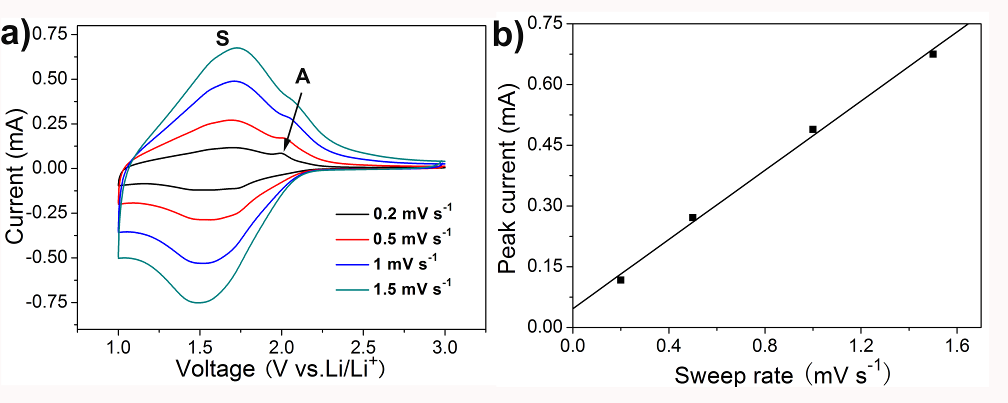


**Figure S8.** (a) Cyclic voltammograms of the TiO_2_-B material after cycling at 10 C for 1000 cycles under varying sweep rates and (b) plot of S-peak currents vs sweep rates.
